# Electrorheology of nanofiber suspensions

**DOI:** 10.1186/1556-276X-6-256

**Published:** 2011-03-25

**Authors:** Jianbo Yin, Xiaopeng Zhao

**Affiliations:** 1 Smart Materials Laboratory, Department of Applied Physics, Northwestern Polytechnical University, Xi'an 710129, China

## Abstract

Electrorheological (ER) fluid, which can be transformed rapidly from a fluid-like state to a solid-like state under an external electric field, is considered to be one of the most important smart fluids. However, conventional ER fluids based on microparticles are subjected to challenges in practical applications due to the lack of versatile performances. Recent researches of using nanoparticles as the dispersal phase have led to new interest in the development of non-conventional ER fluids with improved performances. In this review, we especially focus on the recent researches on electrorheology of various nanofiber-based suspensions, including inorganic, organic, and inorganic/organic composite nanofibers. Our goal is to highlight the advantages of using anisotropic nanostructured materials as dispersal phases to improve ER performances.

## Introduction

Since the discovery of carbon nanotubes (CNTs) by Iijima [1], there has been great interest in the synthesis, characterization, and applications of one-dimensional (1D) nanostructures. Nanofiber is an important class of 1D nanostructures, which offers opportunities to study the relationship between electrical, magnetic, optical, and other physical properties with dimensionality and size confinement. Various nanofibers including metal, inorganic, organic, and inorganic/organic composite have synthesized by different strategies [2-4]. Not only single nanofibers can act as building blocks for the generation of various nanoscale devices such as nanosensors, nanoactuators, nanolasers, nanopiezotronics, nanogenerators, nanophotovoltaics, etc. [5-14], but the incorporation of nanofibers in matrices would also produce advanced composite materials with enhanced properties [4,15-17]. On the other hand, due to some unique characteristics of nanofibers, such as small size, large aspect ratio, thermal, electronic, and transport properties, nanofiber-based suspensions or fluids have also received wide investigations for various applications in thermal transfer, microfluidics, fillers in the liquid crystal matrix, rheological, and biological fields [18-21].

Using external electric or magnetic fields to control the viscosity of fluids or suspensions is very interesting for science and technology because of the potential usage in active control of various devices in mechanical, biomedical, and robotic fields [22-24]. These fluids, whose viscosity can reversibly respond to external electric or magnetic fields, are often referred as 'smart fluids' which include liquid crystal, ferrofluid, magnetorheological (MR) fluid, and electrorheological (ER) fluid. ER fluid consisting of polarizable particles dispersed in a non-conducting liquid is considered to be one of the most interesting and important smart fluids [25,26]. It can be transformed reversibly and rapidly from a fluid-like state to a solid-like state due to the disorder-order transition of particulate phase under an applied external electric field, showing tunable changes in the rheological characteristics. The tunable and quick rheological response to external electric fields makes ER fluid possess potential uses to enhance the electric-mechanical conversion efficiency in mechanical devices such as clutches, valves, damping devices, polishing, ink jet printer, human muscle stimulator, mechanical sensor, and so on [27-29]. In addition, some studies have shown that the ER fluid can be also used to fabricate potentially smart devices in optical, microwave, and sound fields [30-37].

The conventional ER fluid consists of micrometer-size dielectric particles in insulating liquid [25]. Since the ER effect was firstly discovered by Winslow [38], many ER systems including water-containing system such as silica gel, poly(lithium methacrylate), cellulose, and water-free system such as aluminosilicate, carbonaceous, semiconducting polymers have been developed. Some advanced materials including nanocomposites and mesoporous materials have also been investigated for ER fluid applications. The systematic introduction about the progress of ER materials, mechanisms, properties, and applications can be found in several literature reviews at different stages [39-52]. However, the present ER fluids do not possess a versatile performance, and there are still some disadvantages including insufficient yield stress, large particle settling, and temperature instability need to be overcome.

Some recent researches of using nanoparticles as the dispersal phase of ER fluid have led to new interest in the development of non-conventional ER fluid [53-56]. The nanopartile-based ER fluid exhibits extremely high yield strength though its large off-field viscosity and shear stability still need to be improved [57-61]. It is also interesting that compared with the suspension of spherical particles the suspension of 1D nanomaterials has been found to show some enhanced ER or MR effects and even improved dispersion stability recently. The present article provides a general overview on the electrorheology of nanofiber suspensions, including inorganic, organic, and inorganic/organic composite nanofibers.

### Inorganic nanofiber suspensions

Although the effect of particle shape on ER properties has been noted for a long time [62,63], one of the earliest experiments using elongated ER particles was reported by Asano et al. [64,65]. They noted that the suspension containing both spherical and elongated particles produced the largest shear stress under an applied electric field. The suspension consisted of particles made of microcrystalline cellulose particles (The particle sizes were in the range of 20 to 400 μm.) dispersed in silicone oil. From microscopic observation, they suggested that spherical particles had a tendency to adhere to the electrodes, while elongated particles contributed to strengthening the particle chain. Kanu and Shaw [66] studied ER effect of an suspensions containing poly(p-phenylene benzobisthiazole) microfibres with different aspect ratios and found that the storage modulus increased significantly with the increase of aspect ratio. They attributed the increased ER effect to the overlapping of elongated particles and the increased dipolar interactions between elongated particles. Otsubo [67] also studied the effect of particle shape on ER effect by comparing the steady shear viscosity and oscillatory viscoelastic properties of whisker-like aluminum borate suspensions with spherical aluminum borate suspensions. The whisker sample had a diameter of 1 μm and a length of 30 μm, while the diameter of two spherical samples was 2 and 30 μm, respectively. Both steady shear viscosity and oscillatory viscoelastic experiments showed that the whisker suspensions showed a much higher ER response compared to the spherical suspensions at the same volume fraction. It was also found that when the stress amplitude was increased beyond the yield stress, the complex shear modulus of spherical aluminum borate suspensions showed a drastic decline due to the structural rupture. However, the complex shear modulus of whisker suspensions during oscillatory shear showed a shoulder-like decline after the stress exceeded the yield point [68]. The microscopic observation indicated that the fibrous column of whisker-like aluminum borate was thickened after oscillatory shear, which could well explain the enhancement of ER performances. Contrary to the results mentioned above, Qi and Wen [69] observed that the micro-sphere-based suspensions showed better ER performances than micro-rod-based suspensions when the particles had the same diameters. Based on the optical observation of chain-like structure, one possible reason they considered for this was that the micro-rods easily tangled together between the two parallel electrodes, and thus it was difficult for the micro-rods to align well in the direction of the external electric field. The tendency they found for the micro-rod-based suspensions was that the ER effect decreased with the increase of the aspect ratio, while this phenomenon became much weaker in the case when dried particles were substituted for the ones with moisture.

On the other hand, a particle level simulation model was reported recently for investigating the effects of elongated particles on the microstructure and field-induced flow response in the ER fluid [70]. The particles were modeled as a collection of spherical subunits joined by Hookean type connectors, which enabled the modeling of the particle motion through the Newtonian carrier liquid. The simulation results showed that the systems containing elongated particles possessed enhanced stress response when compared with those containing spherical particles at the same volume fraction, and this was similar to that observed from the experiments by Otsubo [67]. Furthermore, it was also pointed out that the stress contribution arising from rotational effects depended on the average orientation vector of the particles at the commencement of the shearing [70]. If the majority of the particles were tilted towards the direction of shearing, a positive contribution to stress would arise as a result of particles rotating against the direction of shearing towards the applied field direction.

Using inorganic nanofibers as the dispersal phase of ER fluid was firstly reported by Feng et al. [71]. In this report, ZnO nanowires were synthesized by thermal evaporation of Zn under controlled conditions without metal catalysts. The mean diameter of the nanowires was about 20 nm. The suspension was prepared by adding 1 g ZnO nanowires into 7 ml silicone oil and then manually stirring for about 30 min. Unlike the usual ER behavior, a decrease in viscosity (negative ER effect) for the ZnO nanowire suspension was observed under DC electric fields. According to the optical microscopic observation, such an anomalous behavior was considered to be due to the occurrence of the electrophoresis migration of ZnO nanowires to two electrodes induced by the electron transfer among ZnO nanowires.

**Figure 1:**
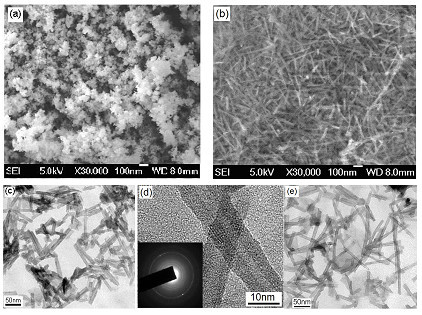
**SEM and TEM images. **SEM images of raw material of titania nanoparticles (a) and formed Na-titanate nanofibers after hydrothermal treatment and 250°C-annealing (b); low-magnification TEM (c) and high-resolution TEM and corresponding ED pattern (d) of Na-titanate nanofibers; (e) TEM image of formed H-titanate nanofibers by washing Na-titanate nanofibers with HCl solution [73].

**Figure 2:**
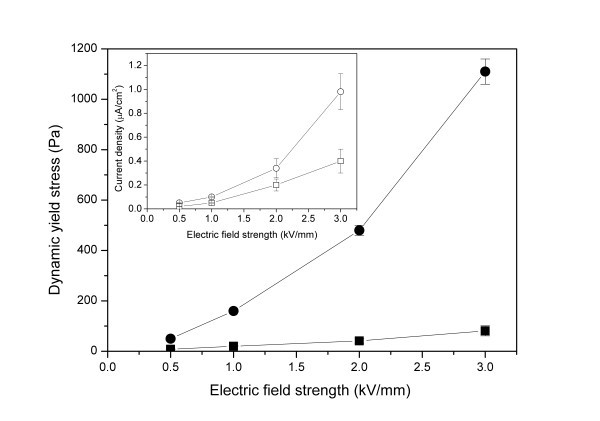
**Yield stress as a function of electric field strength for Na-titanate nanofiber suspension (*solid circle points*) and titania nanoparticle suspension (*solid square points*)**. The *inset *is the corresponding current density of Na-titanate nanofiber suspension (*open circle points*) and titania nanoparticle suspension (*open square point*) [72].

**Figure 3:**
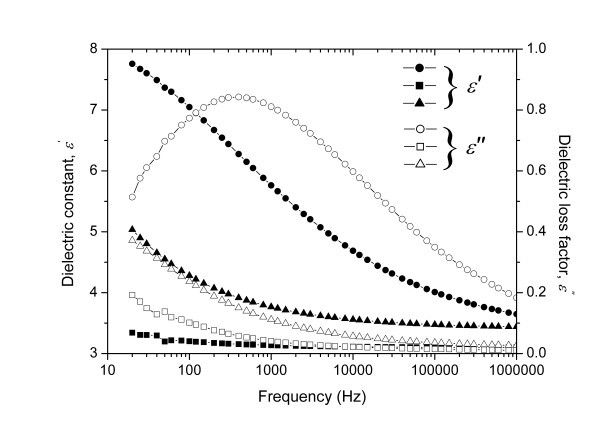
**Dielectric spectra for the suspensions of titania nanoparticles (*square points*), 250°C-heated Na-titanate nanofibers (*circle points*), and 250°C-heated H-titanate nanofibers (*triangle points*) **[73].

A positive ER effect of nanofiber suspensions was reported by the current authors by employing titanate nanofibers as dispersed phase [72,73]. Titanate nanofibers were synthesized by a hydrothermal reaction of titania nanoparticles in high-concentration alkali solution following the Kasuga's report [74]. Titanate nanofibers were uniform nanotube-like morphology with outer diameter of 10 nm and length about 100-200 nm after ultrasonic (see Figure 1). High-resolution transmission electron microscopy (TEM) image (Figure 1d) and selected area electron diffraction (ED) (inset in Figure 1d) showed that the nanotubes consisted of the roll multilayered structure with an inner diameter of 3 nm. The energy-dispersive X-ray spectroscopy analysis showed the titanate nanofibers contained Na, Ti, and O elements. ER properties of suspension of titanate nanofibers in silicone oil were investigated by a steady shear viscosity. Compared to the suspension of titania nanoparticles, the suspension of nanofibers showed higher yield stresses (see Figure 2). At the same time, the alkali-ions intercalated in the interlayer of nanofibers were found to be important to the ER effect of titanate nanofibers. Removal of alkali-ions by acid-treatment did not destroy the nanofiber morphology (see Figure 1e) but weakened ER effect. According to the dielectric spectra analysis (see Figure 3), the decrease of ER effect was considered to be due to the degradation of dielectric property. However, it was noted that the ER effect of nanofiber suspension after removal of alkali-ions was higher than that of pure titania nanoparticle suspension. In particular, after 400°C calcination, the acid-treated nanofibers almost possessed the similar crystal structure and slightly higher dielectric constant compared with pure titania nanoparticles, but the ER effect of the former was still higher than that of the latter. This indicated that the anisotropic nanofiber structure played a role in improving the ER performance. In addition, the ER effect of titanate nanofiber suspension increased with increasing temperatures, which was in accordance with the improving dielectric properties. Another advantage of titanate nanofiber suspension was its lower particle settling rate compared to the conventional granular titania suspension.

In order to investigate the changes of the microstructures of titanate nanofiber suspension under electric fields, the ER behavior of titanate suspension was further measured under oscillatory shear by He et al. [75,76]. Investigation of ER properties by the dynamic oscillation method would be helpful to understand the nature of the interactions among particles forming the internal structures. The results showed that the dynamic moduli of titanate nanofiber suspension were much higher compared to original titania nanoparticle suspension under electric fields. Furthermore, the complex modulus of titanate nanofiber suspension was found to be sensitive to temperature, while that of titania nanoparticle suspension was insensitive at a higher temperature.

Lozano et al. [77] compared the ER effect of Pb_3_O_2_Cl_2 _nanowire, carbon fiber (CNF), and single-walled CNT (SW-CNT) laden suspensions through oscillatory shear experiments in the presence of DC electric fields. It was observed that the CNF suspension developed a negative ER effect in which the storage modulus decreased with the increase of applied electric field. A decrease of 80% in storage modulus was observed at an electric field of 100 V/mm. In the case of the CNT suspension, a similar negative effect was observed. However, the Pb_3_O_2_Cl_2 _nanowire suspension exhibited a positive ER effect and the maximum value was observed at 200 V/mm resulting in an increase of 120% in storage modulus. They considered that the observed negative ER effect in the CNF and CNT suspensions was related to the formation of a layered structure perpendicular to the direction of the electric field rather than a chain-like structure along the electric field direction, which was further due to the difference in electrical conductivity and polarization mechanisms.

**Figure 4:**
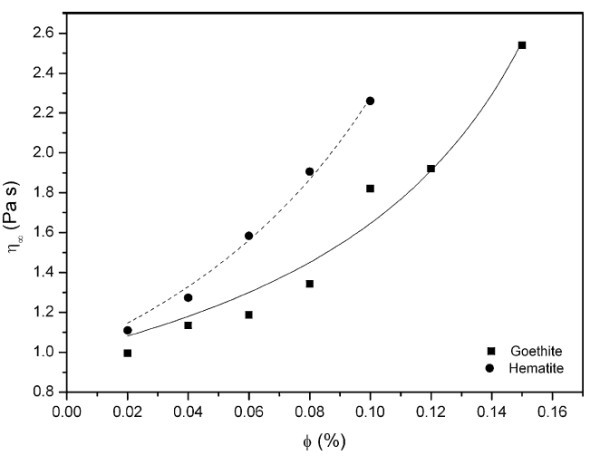
**Viscosity at high shear rate as a function of the particle concentration for goethite and hematite suspensions**. The lines correspond to the fit of the data to the Dougherty-Krieger equation [78].

Ramos-Tejada et al. compared the ER response of the suspension containing goethite (β-FeOOH) nanorods with axial ratio around 8 with the suspension containing polyhedral hematite (α-Fe_2_O_3_) particles with a mean diameter of 105 nm [78]. Both types of particles were said to possess similar chemical compositions and electrical properties and their average particle sizes were very close too. Thus, goethite and hematite samples differed mainly in particle shape. The experiments showed that the goethite suspension changed its rheological behavior from Newtonian without electric field to shear thinning at electric fields. In particular, the suspension of elongated goethite particles produced a more efficient ER response to the electric field than that made of polyhedral hematite particles since the former gave rise to higher yield stress for the same field strength, and exhibited a lower viscosity (see Figure 4) in absence of electric fields. As the chemical compositions and electrical properties, as well as the average particle sizes of elongated goethite and polyhedral hematite were very close, they attributed the ER enhancement to the larger dipole moments induced in elongated particles by the electric field. This consideration also justified why the goethite sample showed the same ER response as hematite one at low electric field of approximately 0.7 kV/mm, while their yield stresses differed significantly at high electric field of 1.5 and 2.0 kV/mm.

**Figure 5:**
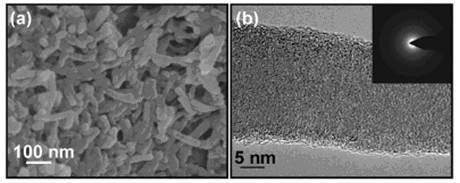
**SEM image (a) and TEM image (b) with the SAED pattern in the inset of the calcium and titanium precipitate nanofibers **[79].

A recent study by Cheng et al. [79] investigated the ER effect of a suspension of calcium and titanium precipitate (CTP) nanofibers. The nanofibers, which were prepared via a precipitation route in an ethanol/water mixed solution system containing tetrabutyl titanate, calcium chloride, oxalic acid dehydrate, had width of 23 nm and length of 40 to 130 nm (Figure 5). The nanofibers were claimed to be polycrystalline, but no clear crystal structure was ascertained according to the electron diffraction pattern. The X-ray diffraction pattern showed that the nanofibers were made of a complex mixture containing calcium oxalate dehydrate, TiOC_2_O_4_(H_2_O)_2_, and TiO(OH)_2_. The rheological measurements showed that the complex nanofibers showed a large yield stress beyond 110 kPa at 66.6 wt% particle concentration in silicone oil, which was about twice higher as high as that of granular suspensions. From the absorption peaks at 3438 and 1649 cm^-1 ^in Fourier transform infrared spectra, however, it could be judged that the nanofiber suspension belonged to a water-containing system. Therefore, the shortages of water effect on ER properties including thermal and electrical instabilities needed to be further overcome for the CTP nanofiber suspension.

Up to now, many kinds of inorganic nanofibers have been prepared by different techniques, but only amorphous or ionic crystal nanofibers can be used as high-performance ER fluids. Furthermore, the disadvantages including the large density and high abrasion of inorganic nanofibers need to be overcome.

### Organic nanofiber suspensions

Due to low density and low abrasion to devices, organic ER systems have been widely investigated in the past decades. Polyelectrolytes and semi-conducting polymers are two kinds of important organic ER systems. In particular, the semi-conducting polymers including polyaniline (PANI), polypyrroles (PPy), poly(p-phenylene) (PPP), polythiophenes, poly(naphthalene quinine radicals) (PNQR), poly(acene quinine radicals) (PANQ), poly(phenylenediamine), oxidized polyacrylonitrile, and their derivatives have been frequently adopted as ER active materials because of the anhydrous character [45,47,49]. The interfacial polarization, induced by the local drift of electron or hole, is believed to be responsible for the ER effect of the semi-conducting polymer systems. By controllable adjustment of π-conjugated bond structure, the conductivity and polarization can be changed.

Among these semi-conducting polymer ER systems, PANI has been considered as one of the most promising alternatives because of its simple preparation, low cost, good thermal stability, and controllable conduction and dielectric properties. Pure PANI and its modifications and composites have been developed for ER application in the past years [80-95]. Studies on these PANI materials greatly help the understanding about ER mechanisms and rheological properties. However, the application of ER fluids based on PANI is still limited to some extent by either low yield stress or particles' sedimentation.

**Figure 6:**
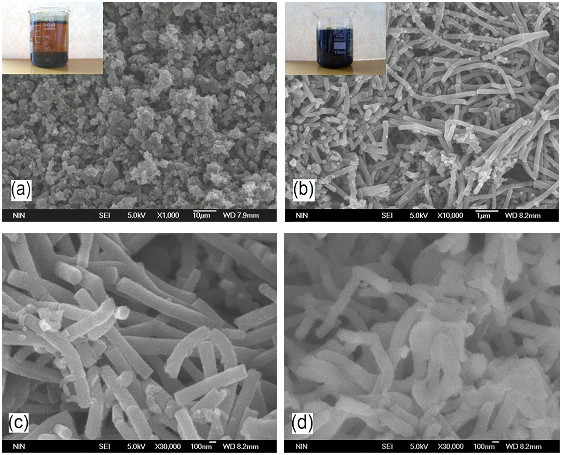
**SEM images of samples**: **(a) **granular PANI, **(b) **PANI nanofibers, **(c) **high resolution SEM images of PANI nanofibers, and **(d) **dedoped PANI nanofibers. The *beakers shown in the insets *contain the resultant granular PANI and PANI nanofiber suspensions, respectively [96].

**Figure 7:**
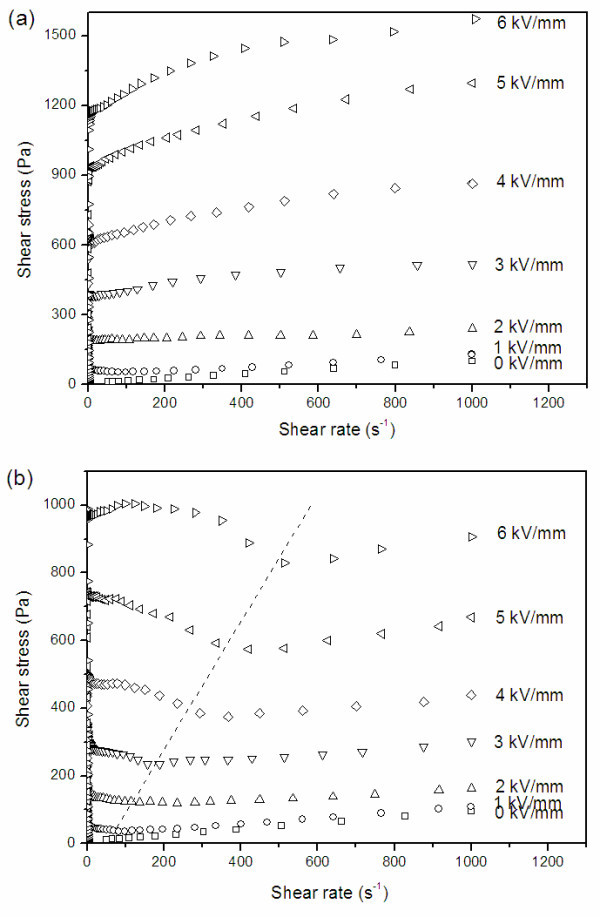
**Shear stress as a function of shear rate for PANI suspensions under different DC electric fields**: **(a) **nanofibers, **(b) **granular. (10 wt%, *T *= 23°C) [96].

Recently, one interesting way was developed to enhance the yield stress by employing nano-fibrous PANI [96]. The PANI nanofibers were easily synthesized on a large scale by an oxidative polymerization of aniline in an acid aqueous solution without mechanical stirring (see Figure 6). The outer diameter was of 200 nm and length of 1 to 5 μm. The BET surface area of PANI nanofibers was 43 m^2^/g, which was higher than that (11 m^2^/g) of granular PANI. After dedoping by immersion in 1 M aqueous ammonia, the PANI nanofibers with decreased conductivity were dispersed into silicone oil with grinding and ultrasonic to form suspensions. Compared to the conventional granular PANI suspension, the nanofiber suspension exhibited larger ER effect. Its shear stress and shear storage modulus were about 1.2 to 1.5 times as high as those of the former. At the same time, the shear stress of the PANI nanofiber suspension could maintain a stable level within the wide shear rate region of 0.1 to 1000 s^-1 ^under various electric fields and the flow curves could be fitted by the Bingham fluid model (see Figure 7a). However, the shear stress of the granular PANI suspension showed a decrease as a function of shear rate to a minimum value, called the critical shear rate (see dot line in Figure 7b), after the appearance of yield stress and then increased again. The flow curves of Figure 7b could not be fitted by the simple Bingham fluid model but could be approximately fitted by the proposed Cho-Choi-Jhon model [97]. These indicated that anisotropic PANI nanofibers not only enhanced the yield stress but also influenced the flow behavior of suspension. In addition, it is interesting that the nanofiber suspension was found to possess better suspension stability compared to the conventional granular suspension when the particle weight fraction was same. No sedimentation occurred for the 15-wt% PANI nanofiber suspension after standing without disturbed for 500 h. This was considered to be related to the small size and large supporting effect of anisotropic nanofibers in suspensions [96].

**Figure 8:**
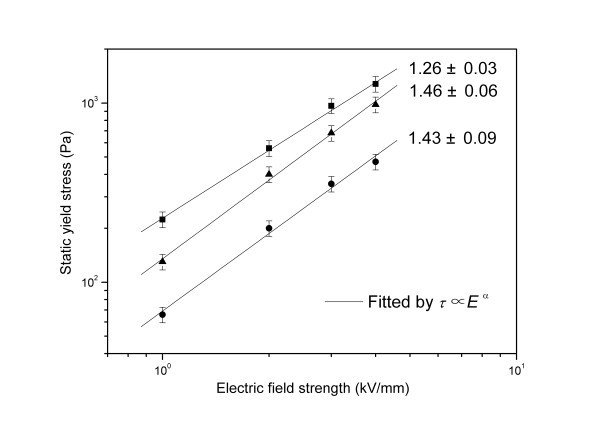
**Static yield stress as a function of electric field strength (15 wt%, *T *= 23°C) for PANI suspensions: nanofibers (*square points*), microparticles (*triangle points*), and nanoparticles (*circle points*) **[98].

By adjusting aniline/acid ratio or solution acidity, not only PANI nanofibers but also spherical micrometer-size and nano-size PANI particles were further prepared by a modified oxidative polymerization in low-cost citric acid solution and their electric, ER, sedimentation, and temperature properties were systematically compared recently [98]. It was found that the PANI nanofiber suspension exhibited the strongest ER effect under electric fields. Its yield stress was about 2.5 to 3.0 times as high as that of the PANI nanoparticle suspension and 1.3 to 1.5 times as high as that of the PANI microparticle suspension. The dependence of yield stress on electric field for the PANI nanofiber suspension was found to follow the power-law relation with a smaller exponent compared with the PANI nanoparticle suspension and microparticle suspension (see Figure 8). This was considered to be related to the anisotropic morphology of PANI nanofibers. The analogical result had also been obtained in the suspensions of spherical and whisker-like inorganic aluminum borate [67,68]. Especially, it was interesting that the PANI nanofiber suspension was found to show lower off-field viscosity compared to the suspension of PANI nanoparticles, which proposed a possible way to overcome the problem of large off-field viscosity of the present nanoparticle-based ER fluids [57-61]. Furthermore, it was found that the PANI nanofiber suspension could maintain a good ER effect in a wide temperature range like the PANI microparticle suspension, while the temperature stability of the PANI nanoparticle suspension was degraded. It was known that the Brown motion disturbed ER structures in nanoparticle suspension systems more easily compared to microparticle suspension systems, but the larger dipole moments and more robust dendrite-like network induced by electric fields in PANI nanofiber suspension were believed to contribute to good temperature stability of ER effect [98].

Very recently, a kind of PPy nanofibers was synthesized for ER fluid application by a chemical oxidative polymerization and a thermo-oxidative treatment [99]. Under electric fields, the PPy nanofiber suspension possessed stronger ER effect than that of the conventional granular PPy suspension at the same volume fraction though the off-field viscosity of the former was lower than that of the latter. It also showed that the thermo-oxidative PPy nanofiber suspension could maintain good ER properties within a wide operating temperature range of 25 to 115°C.

Although organic nanofibers show more advantages in ER properties compared to the conventional granular ones, controlling the morphology of organic nanofibers in the preparation is more difficult compared to inorganic nanofibers. To extend the understanding about the effect of nanofiber morphology on ER properties, it is necessary to synthesize more kinds of organic nanofiber ER materials in the future works.

### Carbonaceous nanofiber suspensions

Carbonaceous material is another very important kind of ER dispersal phase due to its anhydrous character, good ER efficiency, low density, and low electric power consumption. Carbonaceous ER material can be prepared from various organic sources [100-114]. For example, Kojima et al. [103,104] synthesized a kind of carbonaceous ER material composed of condensed polycyclic aromatic compounds with phenyl group and diphenyldiacetylene oligomers by annealing diphenyldiacetylene at an elevated pressure. Choi et al. studied the ER properties of pitch derived coke particles with different oxygen content or crystallographic properties [111]. Dong et al. [114] prepared the carbonaceous ER materials by thermal conversion of fluid catalytic cracking (FCC) slurry. Other carbonaceous materials have also been studied for use as the ER dispersant phase, including carbon black, graphitized carbon particles, carbon cones/disks, and mesoporous carbon [115-118].

**Figure 9:**
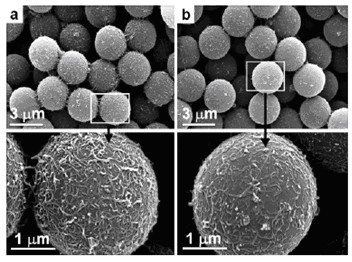
**SEM images of the carbon nanotube-adsorbed PS microspheres using the surfactant**: **(a) **CTAB and **(b) **NaDDBS [120].

CNTs have attracted a lot of scientific interest because of their anisotropic structure and outstanding electrical and mechanical properties for a wide range of applications [119]. In view of the unique characteristics of CNTs, in particular small size, large aspect ratio, thermal, and electronic properties, the ER properties of CNT suspensions have received wide investigations recently. Jin et al. [120] reported for the first time the ER properties of composites consisting of CNTs adsorbed polystyrene (PS) and poly-(methyl methacrylate) (PMMA) microspheres (see Figure 9) when they were dispersed in silicone oil. The microscopic observation showed a clear chain structure formation in the suspension of CNTs adsorbed polymer microspheres when the external electric field was applied. After that, several kinds of composites containing CNTs were further developed by different techniques for ER fluid application [121-128].

Besides adsorbing onto the micospheres for ER fluid application, CNTs have also been added into ER and MR fluids as additives or fillers to decrease the serious particle sedimentation. For example, Fang et al. [129] have introduced SW-CNTs into carbonyl iron (CI) suspension as gap-filler to reduce the sedimentation of CI particles. Li et al. [130] have fabricated the ER fluid comprising nanoparticles/multiwall CNTs (MW-CNTs) composite particles dispersed in silicone oil. This kind of ER fluid displayed dramatically enhanced anti-sedimentation characteristic compared to the ER fluid without MW-CNTs. In the best cases, stabilized suspensions after adding MW-CNTs have been maintained for several months without any appreciable sedimentation being observed. The addition of MW-CNTs was considered to introduce an effective short range repulsive interaction between the ER nanoparticles. However, such repulsive interaction only slightly decreased the yield stress under an electric field.

Although adding CNTs into conventional ER or MR fluids has improved the suspension stability, CNTs only act as fillers or additives in these studies. The alignment and polarizability of pure SW-CNT suspensions under electric fields have been investigated through optical polarimetry by Brown et al. [131]. In the study, a low-frequency alternating-current electric field was applied and the nematic order parameter was determined by measuring changes in the state of polarization of a laser beam transmitted through the suspension. They found that the dependence of the measured alignment of SW-CNTs on the electric field was consistent with a thermal-equilibrium distribution of freely rotating, polarizable rods. The polarizability determined by fitting to this model was consistent with the classical result for a conducting ellipsoid of the dimensions of the nanotube. Recently, Lin et al. [132] further measured the apparent viscosity of a dilute SW-CNT/terpineol suspension under an external electric field. Although the volume fraction of SW-CNTs was very small of 1.5 × 10^-5^, it was experimentally found that the viscosity of suspension increased to more than double at moderate shear rates and electric field of 160 V/mm. In particular, they observed the magnitude of the ER response in the dilute SW-CNT suspension was much higher than that of the conventional suspension containing micro-size glassy carbon spheres at comparable volume fractions. For the suspension of glassy carbon spheres, a suspension of, a three-order-of-magnitude-higher volume fraction must be required to achieve similar increases in the apparent viscosity under the same conditions. The ER response of SW-CNT suspension could be interpreted in terms of an electrostatic-polarization model and the enhanced ER response was attributed to the improved polarization and drag force due to high aspect ratio of the CNTs. Furthermore, the ensemble-averaged particle-orientation angles and apparent shear viscosities of dilute suspensions of SW-CNT/terpineol were also experimentally studied by an optical polarization-modulation method under electric fields during flow recently [133]. Particle-orientation angles for various shear rates (*D*) and electric fields (*E*) were found to collapse when plotted against the parameter, *f *~ *E*^2^/*D *as predicted by the theory developed by Mason and co-workers for the equilibrium orientation angle of ellipsoids under electric fields and shear flow. However, comparison between measured and predicted particle-orientation angles showed poor agreement at intermediate values of *f*. Electrostatic interactions among large-aspect-ratio particles were shown to be significant, and might account for the discrepancy between the measurements and classical theories for even dilute suspensions of nanotubes under both shear and electric fields. Under DC electric fields, however, the CNT suspension showed a negative ER behavior due to large electrical conductivity [77].

The CNT suspensions mentioned above are made of the commercial CNTs, their yield strength or ER efficiency is too low to be used in many ER devices and the electrical breakdown easily occurrs in these suspensions containing commercial CNTs because of the easy percolation of pseudo-1D conductivity [77,132].

**Figure 10:**
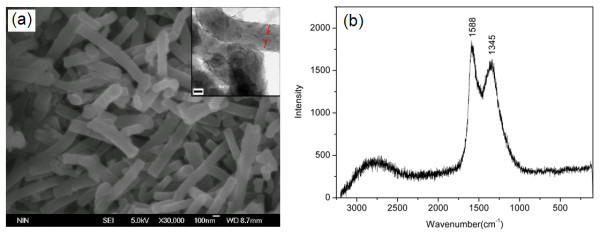
**The morphology and Raman spectra of N-CTs.** (a) SEM image and TEM image (*inset*, scale bar = 50 nm) of N-CTs, (b) Raman spectra of N-CTs [135].

**Figure 11:**
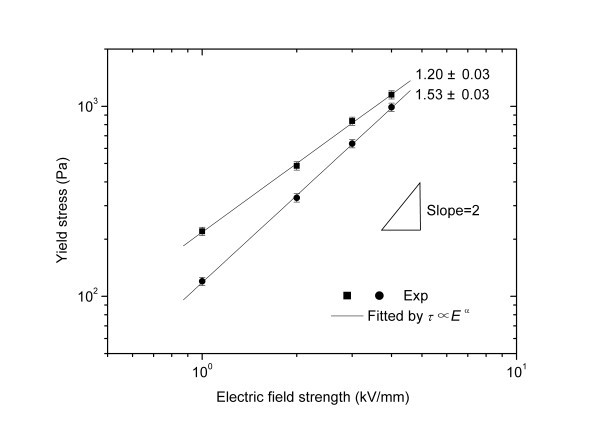
**Yield stress as a function of electric field strength for N-CT suspension (*square symbol*) and heat treated granular PANI suspension by the same process (*circle symbol*) (*T *= 23°C, 15 vol.%) **[134].

**Figure 12:**
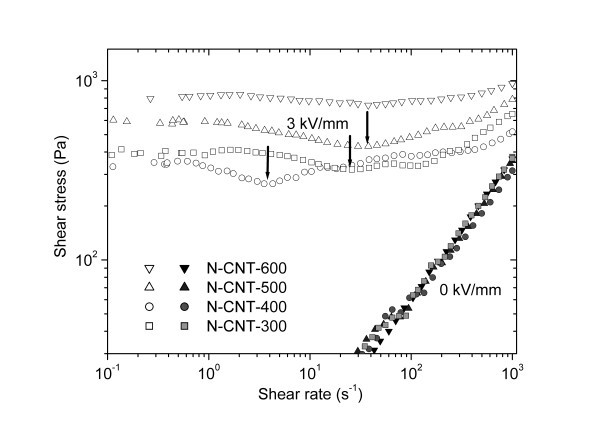
**Flow curves of shear stress vs. shear rate for N-CT suspensions under zero (*solid symbol*) and 3 kV/mm (*open symbol*) electric fields (*T *= 23°C, 15 vol.%) **[134].

Very recently, a kind of nanotube-like nitrogen-enriched carbonaceous nanofibers (N-CTs) were prepared by the heat treatment of conducting PANI nanofibers and then were used as new carbonaceous ER materials [134]. The heat treatment temperature was found to be important to obtain N-CTs with the optimal ER effect. The heat treatment at the temperature lower than 500°C easily transformed PANI nanofibers into thermally degraded PANI nanofibers whose conductivities were too low to induce a strong ER effect, while the heat treatment at temperature higher than 600°C transformed PANI nanofibers into the partially graphitized nitrogen-containing nanotubes whose conductivities were too high to finish ER measurements because of the electrical short circuit. When PANI nanofibers were treated in vacuum at the temperature range of 500 to 600°C, the obtained N-CTs were suitable to be used as ER dispersal phase because they had the moderate conductivity. After heat treatment, the nanofiber morphology was found to be well preserved except that the diameters showed shrinkage and the aspect ratio of nanotubes slightly decreased with increasing heat treatment temperatures [134]. Figure 10 showed the morphology and Raman spectra of N-CTs obtained at 550°C. The N-CTs possessed the uniform nanotubular morphology with a diameter of 90 to 150 nm and a length of 1 to 2 μm. The Raman spectra of the N-CTs showed two broad bands centered at about 1588 cm^-1 ^(G band) and 1345 cm^-1 ^(D band), characteristic of amorphous carbon or disordered graphites. The N-CTs mainly contained C (77.5 wt%), N (12.6 wt%), and other elements (such as H and O). These indicated that the heat treatment at 550°C had transformed the PANI nanofibers into the amorphous nitrogen-enriched carbonaceous nanotubes [135]. Under electric fields, the rheological results showed that the N-CT suspension possessed versatile ER performance including high ER efficiency, good dispersion stability, and temperature stability. Especially, compared to the corresponding suspension of heat treated granular PANI, the N-CT suspension showed better dispersion stability and higher ER effect (see Figure 11). The analogical result was also observed in the dilute ER fluid containing commercial CNTs [132]. When a power-law relation *τ*_y _∝ *E*^α ^was used to fit the correlation of yield stresses and electric fields, it was also found that the exponent of the N-CT suspension was smaller than that of granular suspension. This was mainly related to the particle morphology because other factors such as particle concentration, particle's conductivity, liquid phase, and so on were the same for N-CTs and heat treated granular PANI. The similar result was also observed in the PANI nanofiber suspension [96,98] and in the whisker-like inorganic aluminum borate suspension [67]. Furthermore, the ER effect of N-CT suspension could be adjusted by varying heat treatment temperatures and the N-CTs obtained at around 600°C exhibited the maximum ER effect (see Figure 12). This was explained by the polarization response, which originated from the regular change of conductivity of N-CTs as a function of heat treatment temperatures [134]. It showed that under electric fields the N-CT suspension showed good temperature stability in ER effect though its off-field viscosity decreased with elevated temperatures. Meanwhile, the flow curve of shear stress vs. shear rate also maintained a stable level and the critical shear rate shifted toward high values as the operating temperature increased. The dynamic viscoelastic measurement showed that the storage modulus slightly increased with increasing operating temperature, also confirming the good temperature stability of ER effect of N-CT suspension. The dielectric spectra of N-CT suspension and the dielectric parameters calculated by the Cole-Cole equation could explain the temperature dependence of ER effect of N-CT suspension [135].

The field response of vapor-grown carbon nanofibers (VGCFs) was also observed when dispersed in polydimethylsiloxane [136]. It was found that a DC electric or magnetic field was applied to induce the formation of an aligned structure. Upon application of a DC electric field, an aligned ramified network structure of VGCFs developed between the electrodes. In the formation of the network structure, ends of VGCFs became connected to ends of other VGCFs, which were followed by rotation and orientation of the VCGFs. On the other hand, upon application of a magnetic field, the VGCFs were only rotated, without the formation of a network. The viscosity of the polydimethylsiloxane matrix was found to influence the structural formation process. However, no rheological data were reported in the VGCFs/polydimethylsiloxane suspension.

Although 1D carbonaceous material is potential as novel nanofiber ER fluids, it should point out that the suspension durability or dispersion stability is still a challenge due to the facile aggregation of 1D carbon nanomaterial. One feasible way of improving dispersion stability is to prepare the polymer graft 1D carbonaceous material by the graft reaction of carboxyl groups on the carbon material [137].

### Inorganic/organic composite nanofiber suspensions

Although the inorganic and organic ER materials show many advantages, the disadvantages of single component are also prominent and difficult to be harmonized. To obtain ER fluids with comprehensive performances, the fabrication of composite ER particles have been proposed because they can combine the advantages of different components. The most popular composite ER particles are core/shell structured particles [138-142]. On one hand, the particle sedimentation problem of ER fluids is expected to be overcome by using low density polymer or hollow sphere as core. On the other hand, it is considered to be feasible to increase ER effect by adjustably controlling the conductivity and dielectric constant of core and shell. The detailed theorized investigations have included various core/shell composite particles [143-147]. It has indicated that promising ER fluids for using over a wide frequency range were those which contained highly conducting particles coated with an insulating shell having high dielectric constant and high electric breakdown strength.

**Figure 13:**
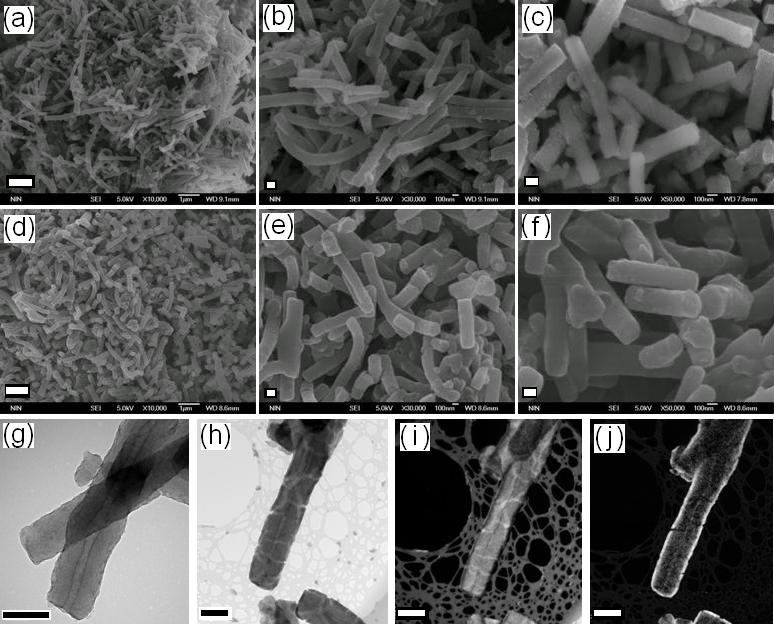
**SEM images: as-made PANI nanofibers (a to c) and PANI@titania nanofibers (d to f)**. (The *scale bar *is 1 μm for **(a) **and **(d)**, 100 nm for **(b)**, **(c)**, **(e)**, and **(f)**). TEM images: as-made PANI nanofibers **(g) **and PANI@titania nanofibers **(h)**. EELS analysis of PANI@titania nanofibers for C element **(i) **and Ti element **(j)**. (The *scale bar *is 200 nm for **(g) **to **(j)**) [148].

Having considered the advantages of core/shell composite ER particles, researchers have paid significant attention to inorganic/organic composite nanofibers for use as promising ER fluids recently [148-152]. By combining conducting polymer nanofibers and insulating inorganic dielectric, a kind of well-organized coaxial cable-like PANI@titania nanofibers was synthesized by a facile hydrolysis of tetrabutyl titanate in the presence of conducting PANI nanofibers for ER fluid application [148]. From Figure 13, it was noted that the PANI nanofiber core had a diameter of 150 to 200 nm and length of 0.5 to 3.0 μm. The thickness of sheath layer was about several tens of nanometers, depending on the amount of water used in the reaction. The sheath thickness increased with the increase of the amount of water. In the coating process of titania sheath, the chemical structure of PANI nanofibers was less changed because of no additional acid or alkali was added and thus the physical properties of PANI core was expected to maintain unchanged. Under electric fields, the suspension of PANI@titania nanofibers exhibited the lightly smaller yields stress compared to that of the suspension of pure dedoped PANI nanofibers, but its leaking current density was significantly lower than that of the latter. This was attributed to a sufficient electrical insulating effect of titania sheath to conducting PANI core (the dielectric constant of the PANI@titania nanofiber suspension was about 5.0 at 1 kHz) and thus the coaxial cable-like PANI@titania nanofibers could be used as a potential dispersal phase of ER fluid with low electric power consumption.

**Figure 14:**
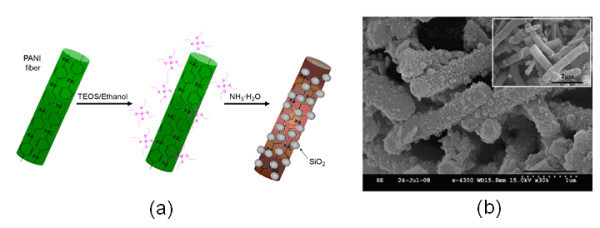
**Silica-PANI fibers.** (a) Schematic diagram of synthetic process of silica-PANI fibers and (b) SEM images of resulted silica-PANI fibers (*inset*: PANI fibers) [149].

A silica nanoparticle decorated PANI nanofibers were also successfully synthesized as a dispersed phase of an ER fluid recently [149]. In this study, the PANI fibers obtained through interfacial polymerization were about 300 to 400 nm in diameter and 2 to 5 μm in length. Then the fibers were redispersed in ethanol containing tetraethyl orthosilicate (TEOS), and silica nanoparticles were formed on the surface of the fibers through a modified Stöber method (see Figure 14). Due to the use of the hydrous ammonia in the synthesis, however, the PANI fiber core was the dedoped emeraldine base-form in the resulted silica nanoparticle decorated PANI fibers. The ER properties of the suspensions based on pure PANI fibers and silica-PANI fibers were compared using a rotational rheometer under electric fields, demonstrating lower shear stress and slight different flow curves for the silica decorated PANI fiber suspension.

On the other hand, the composite nanofibers composed of inorganic core coated by organic sheath were also developed for ER fluid application. Cheng et al. [150] have synthesized PANI/titanate composite nanofibers by in situ chemical oxidative polymerization directed by block copolymer. In their preparation, the inorganic titanate nanofibers were modified first by block copolymer and then PANI was coated by chemical oxidative polymerization of aniline monomer in the presence of modified titanate nanofibers. Although the authors did not give a comparison of ER effect of the composite nanofibers with the single core or shell component, it was found that the ER activity of PANI/titanate nanofiber suspension varied with the ratio of aniline to titanate. In particular, the PANI/titanate nanofiber suspension was found to show a higher ER effect than that of the sphere-like PANI/titania nanoparticle suspension, indicating a positive contribution to ER enhancement from the anisotropic morphology. The ER enhancement was interpreted by the dielectric spectra analysis; a larger dielectric loss enhancement and a faster rate of interfacial polarization were responsible for the higher ER activity of the PANI/titanate nanofiber-based suspension. It should be pointed out that, different from the cable-like PANI@titania nanofibers mentioned above, the PANI/titanate composite nanofibers must be dedoped to decrease the conductivity of PANI sheath before they were used as ER dispersal phase. Otherwise, the suspensions will subject to an electrical short circuit under high electric fields. Compared to those composed of organic core coated with inorganic sheath, however, this kind of inorganic/organic composite nanofibers possessed an advantage of low abrasive action to devices.

Core/shell composite nanofibers can act as the model materials to match the advantages of different components for the optimal ER performances, but the wreck of coating layer under high shearing force is still a problem to limit their practical applications. The formation of inorganic/organic hybrid composite nanofibers provides an alternative way. Due to the stronger interaction between inorganic and inorganic components, the hybrid composite nanofibers are expected to possess more stable mechanical properties when the suspensions subjected to strong shearing flow. For example, a kind of conducting PPy nanofibers with TiO_2 _nanoparticles was synthesized in the presence of β-naphthalenesulfonic acid by chemical oxidative polymerization recently [151]. The results indicated that the structural and electrical properties of the composite nanofibers were influenced by the content of TiO_2 _nanoparticles. The DC conductivity of the composite nanofibers increased by one order of magnitude when the concentration of TiO_2 _was 0.1 M compared with pure PPy nanofibers. The AC conductivity of the composite nanofibers showed the similar trend with the TiO_2 _content and obeyed the power law index in the 10 to 10^7^-Hz range. The ER properties of the composite nanofibers in silicone oil were also evaluated under steady and oscillatory shear. Chuangchote et al. used an electrospinning method to fabricate mats of nanofibers from neat and carbon black (CB) nanoparticle-loaded poly(vinyl alcohol) (PVA) solutions in reverse osmotic water. The ER properties of the as-spun neat and CB-loaded PVA fiber mats with the average diameter of the individual fibers being about 160 nm and the thickness of the mats being about 20 to 30 μm were characterized [152]. While their Young's modulus was found to increase, both the tensile and the elongation at break of the as-spun fiber mats were found to decrease, with the addition and increasing amount of CB. With or without the electrostatic field, both the storage and the loss moduli for all of the as-spun neat and CB-loaded PVA fiber mats were found to increase with increasing frequency. Under the electrostatic field, the dynamic mechanical responses were found to increase with initial increase in the applied electrostatic field strength (EFS) and level off at a certain applied EFS value. At the applied EFS value of 100 V/mm, the dynamic mechanical responses were found to increase with the initial increase in the CB content and level off when the CB content was greater than about 6%. However, no viscosity properties were studied for the CB-loaded PVA naonfibers when dispersed in insulating liquid.

## Conclusions

The preparation of non-conventional ER fluids based on nanoparticles is an area of growing interest from both the fundamental and application points of view. In this review, we have summarized recent researches in the synthesis and ER properties of nanofiber suspensions including inorganic, organic, and inorganic/organic composite nanofibers. Although Qi and Wen [69] have observed that the microsphere based suspensions showed high yield stress than that of micro-rod based suspensions when the particles had the same diameters, most of researches have indicated that the small size and anisotropic structure with large aspect ratio played a great role in improving the suspension stability and ER properties of nanofiber suspensions compared to the conventional sphere suspensions. Some nanofiber suspensions have also been found to show lower off-field viscosity compared to nanosphere suspensions, which provides a possible way to solve the problem of large off-field viscosity of present nanoparticle based ER fluids. Especially, it should be noted that the theoretical and experimental investigations performed recently on MR fluids also showed that suspensions containing magnetic fibers or nanofibers gave rise to an enhanced magnetorheology when compared with conventional MR fluids made up of spherical particles [153-163]. Therefore, it is reasonable to point out that employing anisotropic nanostructured particles to improve ER performances is a very interesting topic. However, the disadvantages including complicated preparation process, nanofiber aggregation, etc. and the further understanding about physical and chemical mechanisms behind the electrorheology of nanofiber suspensions need to be noted in the future works. In addition, the exploration of the nanofiber ER suspensions for new applications in advanced sensors and actuators in MEMS and biotechnology fields should be noted.

## Abbreviations

CB: carbon black; CNF: carbon fiber; CNTs: carbon nanotubes; CTP: calcium and titanium precipitate; ER: electrorheological; MR: magnetorheological; MW-CNTs: multiwall CNTs; PANI: polyaniline; PANQ: poly(acene quinine radicals); PNQR: poly(naphthalene quinine radicals); PPP: poly(p-phenylene); PPy: polypyrroles; PVA: poly(vinyl alcohol); SW-CNTs: single-walled CNT; VGCFs: vapor-grown carbon nanofibers.

## Competing interests

The authors declare that they have no competing interests.

## Authors' contributions

Both authors contributed equally. Both authors read and approved the final manuscript.
